# Association of meteorological factors with the frequency of primary rhegmatogenous retinal detachment in Japan

**DOI:** 10.1038/s41598-021-88979-x

**Published:** 2021-05-05

**Authors:** Masanobu Iida, Hiroshi Horiguchi, Satoshi Katagiri, Yuka Shirakashi, Yuki Yamada, Hisato Gunji, Tadashi Nakano

**Affiliations:** Department of Ophthalmology, The Jikei University School of Medicine, 3-25-8 Nishi-shimbashi, Minato-ku, Tokyo, 105-8461 Japan

**Keywords:** Diseases, Medical research, Risk factors

## Abstract

This 5-year ecological study assessed the association between meteorological factors and rhegmatogenous retinal detachment (RRD) frequency in 571 eyes of 543 cases of primary RRD at the Jikei University Kashiwa Hospital, Japan. We examined the monthly and seasonal distributions of RRD frequency using one-way analysis of variance. We then evaluated the relationship between monthly RRD frequency and 36 meteorological parameters using Poisson regression analysis. Furthermore, we developed multivariate regression models to predict the frequency of RRD based on specific meteorological parameters. There were no significant differences in the monthly and seasonal distributions (monthly, P = 0.99; seasonal, P = 0.77). The following eight parameters were associated with a lower RRD frequency: average sea level barometric pressure and average daily variation of average temperature, maximum temperature, maximum wind speed, maximum instantaneous wind speed, humidity, average sea level barometric pressure, and minimum sea level barometric pressure (P < 0.05). The best model to predict RRD frequency showed sufficient validity (Akaike’s information criterion with correction for small sample size = 332.0) and predictive power (proportion of variance explained by cross-validation method = 84.82%, 95% CI 72.18–93.72). In conclusion, low atmospheric pressure and high meteorological stability are significantly associated with a higher frequency of RRD. In addition, the Poisson regression analysis showed sufficient validity and predictability for predicting RRD frequency.

## Introduction

The retina is a thin, multi-layer tissue located at the back of the eyeball; it is composed of 10 layers from the inner limiting membrane to the retinal pigment epithelium^1^. When humans see an image, the retina captures the image and converts it into signals, which are then sent to the brain^2^. Retinal detachment (RD) is a condition in which the retina is pulled away from its normal position and is defined as the separation of the neurosensory retina from the retinal pigment epithelium^3,4^. When RD occurs due to round holes, tears, or breaks in the retina, the RD is classified as rhegmatogenous RD (RRD)^3^.

RRD is one of the most serious and major ophthalmological diseases, which can lead to irreversible loss of vision in the absence of appropriate treatment^5,6^. The annual RRD incidence varies across different ethnicities and geographical regions, ranging from 6.3 to 18.2 cases per 100,000 persons, as reported in studies conducted in both Western and Asian countries^7–9^. An epidemiological study from Japan reported an RRD incidence of 16.5 (21.9 in male and 11.7 in female patients) per 100,000 persons with a mean patient age of 54.4 years^10^.

Multiple factors can influence RD occurrence; these include myopia, cataract surgery, trauma, intraocular inflammation, retinal breaks, and vitreoretinal disorders^11,12^. Researchers have also studied the association between meteorological factors and RD frequency by analyzing its monthly and seasonal variations^13–21^. These studies have focused on identifying meteorological parameters that may have a significant effect on RD frequency^15,16,18–22^. Although several such studies have been conducted across the globe, the association between meteorological factors and RD frequency remains controversial^23,24^. Moreover, no similar studies have been conducted in Japan.

Herein, we first investigated the association between meteorological factors and RD frequency in Japan. Additionally, multivariate regression models with varying numbers of meteorological parameters were developed to predict RRD frequency.

## Methods

### Ethical approval

This was a retrospective ecological study, approved by the Institutional Review Board of the Jikei University School of Medicine and was conducted in accordance to the principles of the Declaration of Helsinki [approval number: 32–224 (10305)]. According to the Ethical Guidelines for Medical and Health Research Involving Human Subjects (the Japanese Ministry of Health, Labour and Welfare), it was not necessary to get informed consent from each research subject as this study did not involve intervention and neither utilized human biological specimens nor special care-required personal information. Instead, we posted the documents approved by the Institutional Review Board of the Jikei University School of Medicine on the website and announced the information of this research on the bulletin board in our hospital. We guaranteed the subjects the right to refuse participation in this study at any time. The parents/legally authorized representatives of subjects that are under age of 20 were also guaranteed the right to refuse.

### Patient eligibility criteria

We reviewed the medical records of patients who were diagnosed with primary RRD at the Department of Ophthalmology, the Jikei University Kashiwa Hospital, Kashiwa, Japan. The study period was defined as January 2015 to December 2019, using the date of surgical treatment. Since almost all patients with RRD underwent surgery in our hospital, only a few patients with RRD with no surgical history were excluded from the analysis. We also excluded patients diagnosed with secondary RRD (traumatic, tractional, and iatrogenic RD) and those with primary RRD if an accurate day of onset (within a 1-week range) could not be determined. Patient characteristics in the medical records included age, sex, and day of RRD onset. We defined day of onset as the day when the first RRD-related symptom (e.g., floaters, visual field defect, distortion, blurred vision, photopsia, and decline of visual acuity) appeared. Instances of recurrent RRD in the same patient were counted as additional cases.

### Meteorological data

We obtained data on 12 daily meteorological parameters from the Japan Meteorological Agency (https://www.data.jma.go.jp/gmd/risk/obsdl/index.php), namely average temperature (°C), maximum temperature (℃), minimum temperature (℃), amount of precipitation (mm), duration of sunshine (h), average wind speed (m/s), maximum wind speed (m/s), and maximum instantaneous wind speed (m/s) in Abiko City, and average vapor pressure (hPa), humidity (%), average sea level barometric pressure (hPa), and minimum sea level barometric pressure (hPa) in Chiba City. These locations (Abiko and Chiba) were the nearest observation sites to Kashiwa city, for which meteorological data were available.

### Statistical analysis

We investigated the monthly and seasonal variations in RRD frequency (eyes per month/eyes per season). For the analysis, a year was divided into four seasons: spring (March, April, and May); summer (June, July, and August); autumn (September, October, and November); and winter (December, January, and February). Monthly and seasonal comparison of RRD frequency was performed using one-way analysis of variance with Python 3.7.6. Values of P < 0.05 were considered statistically significant.

Subsequently, we predicted RRD frequency (eyes per month) from meteorological parameters with a Poisson regression model, using Python 3.7.6 and MATLAB 9.1. First, we calculated RRD frequency and average meteorological parameters for each month. In addition, we reconstituted monthly variation (MV) and average daily variation (ADV) of each month for each meteorological parameter. MV was calculated as the difference between the parameters for the current and previous months. ADV for each day was calculated as the difference between the parameters for the current and previous days. We used the absolute value to calculate variation in order to avoid canceling values. The monthly RRD frequency was used as the response variable and the average meteorological parameters with two types of variation parameters were used as the explanatory variables. We conducted a univariate analysis for each parameter pair, and those with P < 0.05 were considered statistically significant.

Lastly, we performed a multivariate analysis, using three types of multivariate regression models: Models A, B, and C; Model A included all 36 parameters as explanatory variables; Model B included parameters that were significantly correlated (P < 0.05) with RRD frequency in the univariate analysis; Model C, in which the effect of multicollinearity was excluded, did not include the significantly correlated explanatory variables from Model B if the variance inflation factor (VIF) > 5. In each model, we calculated the Akaike’s information criterion with correction for small sample size (AICc), Bayesian information criterion (BIC), proportion of variance explained (VE), and proportion of variance explained by cross-validation method (CV-VE)^25–27^. For the calculation of CV-VE, the 5-year data were randomly divided into training (80%) and test (20%) data. We calculated VE by substituting test data into the model created using the training data, which was then defined as CV-VE. Furthermore, we applied the leave-one-out cross-validation method to determine the validity of each model.

## Results

### Patient demographics

In total, medical records of 571 eyes in 543 primary RRD cases were obtained for the study, among which there were 185 women (34.1%) and 358 men (65.9%). The average patient age was 56.10 ± 13.67 (15–92) years. Further, 322 (56.4%) right eyes and 249 (43.6%) left eyes were included in the analysis, and there were 28 (4.9%) cases of recurrent RRD.

### RRD frequency (monthly and seasonal distribution)

**Figure 1:**
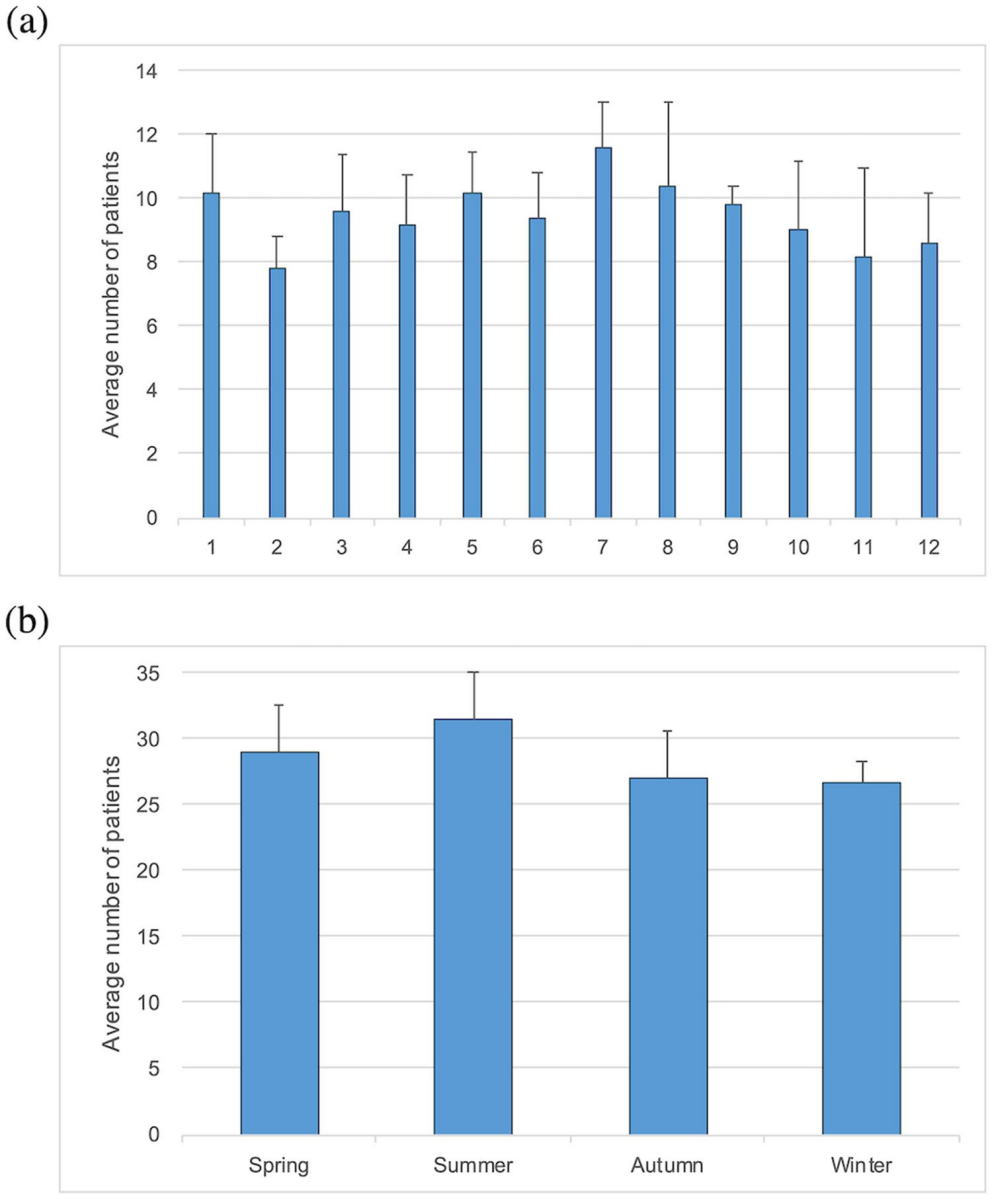
Distribution of rhegmatogenous retinal detachment frequency. (**a**) Monthly distribution, with months indicated on the horizontal axis. (**b**) Seasonal distribution. (**a**,**b**) There are no significant differences among monthly and seasonal variations. The average annual frequency of rhegmatogenous retinal detachment is indicated on the vertical axis.

The monthly RRD frequency (eyes per month) was highest in July (n = 11.6) and the lowest in February (n = 7.8) (Fig. 1a). The seasonal RRD frequency (eyes per season) was the highest in summer (n = 31.4) and lowest in winter (n = 26.6) (Fig. 1b). However, there were no statistically significant differences within the monthly and seasonal distributions (monthly, P = 0.99; seasonal, P = 0.77).

### Univariate Poisson regression analysis

Using univariate analyses, the following eight parameters were found to be associated with a lower RRD frequency (eyes per month): average sea level barometric pressure, average daily variation of average temperature, maximum temperature, maximum wind speed, maximum instantaneous wind speed, humidity, average sea level barometric pressure, and minimum sea level barometric pressure (P < 0.05) (Table 1). In other words, as the average sea level barometric pressure decreased and stability of the seven parameters increased, the frequency of RRD significantly increased.

**Table 1:** Univariate analysis of 36 meteorological parameters and frequency of rhegmatogenous retinal detachment.

	Average meteorological parameters			Monthly variation			Average daily variation		
	Coefficient	P-value	95% CI	Coefficient	P-value	95% CI	Coefficient	P-value	95% CI
Average temperature (℃)	0.0080	0.141	− 0.003 to 0.019	0.0045	0.650	− 0.015 to 0.024	− 0.2845	0.012	− 0.506 to − 0.063
Maximum temperature (℃)	0.0089	0.120	− 0.002 to 0.020	0.0046	0.646	− 0.015 to 0.024	− 0.1780	0.028	− 0.337 to − 0.019
Minimum temperature (℃)	0.0072	0.151	− 0.003 to 0.017	0.0048	0.610	− 0.013 to 0.023	− 0.1283	0.090	− 0.277 to 0.020
Amount of precipitation (mm)	− 0.0037	0.792	− 0.032 to 0.024	0.0048	0.656	− 0.016 to 0.026	0.0003	0.971	− 0.017 to 0.018
Duration of sunshine (h)	− 0.0010	0.976	− 0.066 to 0.064	− 0.0006	0.981	− 0.051 to 0.050	− 0.0514	0.350	− 0.159 to 0.056
Average wind speed (m/s)	− 0.0506	0.680	− 0.291 to 0.190	− 0.1439	0.290	− 0.411 to 0.123	− 0.2876	0.167	− 0.696 to 0.121
Maximum wind speed (m/s)	− 0.0580	0.382	− 0.188 to 0.072	− 0.0713	0.307	− 0.208 to 0.065	− 0.2320	0.017	− 0.446 to − 0.061
Maximum instantaneous wind speed (m/s)	− 0.0490	0.182	− 0.121 to 0.023	− 0.0252	0.455	− 0.091 to 0.041	− 0.1312	0.008	− 0.423 to − 0.041
Average vapor pressure (hPa)	0.0078	0.133	− 0.002 to 0.018	0.0057	0.538	− 0.012 to 0.024	− 0.0633	0.372	− 0.229 to − 0.034
Humidity (%)	0.0019	0.640	− 0.006 to 0.010 s	− 0.0040	0.469	− 0.015 to 0.007	− 0.0252	0.039	− 0.202 to 0.076
Average sea level barometric pressure (hPa)	− 0.0232	0.036	− 0.045 to − 0.002	− 0.0155	0.257	− 0.042 to 0.011	− 0.0931	0.002	− 0.049 to − 0.001
Minimum sea level barometric pressure (hPa)	− 0.0221	0.094	− 0.048 to 0.004	− 0.0067	0.624	− 0.033 to 0.020	− 0.0883	0.001	− 0.152 to − 0.034

*CI* confidence interval.

### Multivariate Poisson regression analysis

Three models of multivariate analyses to predict RRD frequency (eyes per month) showed that prediction accuracy improved by reducing the parameters (Table 2). Model A, which used all 36 variables, had the following values: AICc = 472.4, BIC = 422.1, VE = 96.0%, and CV-VE =  − 73.86% (95% CI − 625.61–83.96%). In contrast, Model B, which used only eight significantly correlated variables, had the following values: AICc = 337.4, BIC = 352.6, VE = 89.1%, and CV-VE = 83.8% (95% CI 70.72–93.16%), with a marked improvement in CV-VE. Among the eight significantly correlated parameters, two pairs showed multicollinearity: maximum instantaneous wind speed (ADV) and maximum wind speed (ADV; VIF = 5.6, r = 0.91), and minimum sea level barometric pressure (ADV) and average sea level barometric pressure (ADV; VIF = 9.33, r = 0.94). After removal of maximum wind speed (ADV) and average sea level barometric pressure (ADV) used in Model B, Model C included the remaining six parameters as explanatory variables: average sea level barometric pressure, average daily variation of average temperature, maximum temperature, maximum instantaneous wind speed, humidity, and minimum sea level barometric pressure. Model C had the following values: AICc = 332.0, BIC = 344.5, VE = 89.0%, and CV-VE = 84.82% (95% CI 72.18–93.72%), with improved AICc, BIC, and CV-VE as compared to Model B. According to model C, the predicted RRD frequency was calculated using the following formula:

**Table 2:** Three types of multivariate regression models.

Model	Variables	AICc	BIC	VE	CV-VE (95% CI)
Model A	All variables (n = 36)	472.37	422.05	96.03%	− 73.86% (− 625.61 to 83.96%)
Model B	Variables with P < 0.05 in univariate analysis (n = 8)	337.38	352.63	89.05%	83.76% (70.72 to 93.16%)
Model C	Correlated variables in Model B were removed (n = 6)	331.97	344.48	89.04%	84.82% (72.18 to 93.72%)

*AICc* Akaike’s information criterion with correction for small sample size, *BIC* Bayesian information criterion, *CI* confidence interval, *CV-VE* proportion of variance explained by cross-validation method, *VE* proportion of variance explained.

$$\text{ln}\left(predicted \,RRD\, incidence\left[eyes \,per \,month\right]\right)=\left(- 0.011\right)\times \left(station\, barometric\, pressure\left[hPa\right]\right)+\left(- 0.002\right)\times \left(ADV\, of \,average\, temperature\left[^\circ C\right]\right)+\left(- 0.117\right)\times \left(ADV \,of \,maximum \,temperature\left[^\circ C\right]\right)+\left(- 0.033\right)\times \left(ADV\, of\,maximum \,instantaneous \,wind\, speed\left[\frac{m}{s}\right]\right)+0.035 \times \left(ADV\, of \,humidity\left[\%\right]\right)+\left(- 0.102\right)\times \left(ADV \,of \,minimum \,sea \,level \,barometric \,pressure\left[hPa\right]\right)+13.594$$

**Figure 2:**
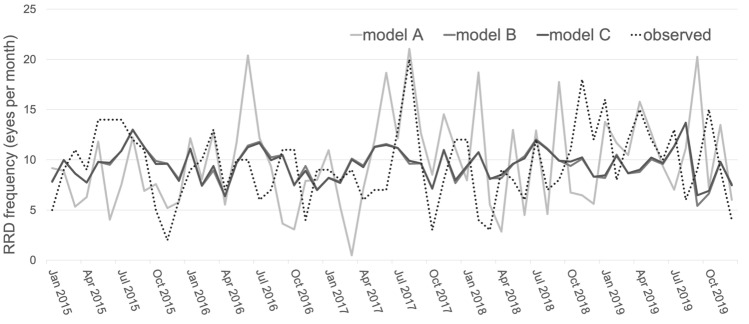
Time course epidemic curves for multivariate regression models. The observed rhegmatogenous retinal detachment (RRD) frequency is shown by the black dashed line. RRD frequency predicted using the leave-one-out cross-validation method in Model A (using all 36 meteorological parameters), Model B (using eight meteorological parameters), and Model C (using six meteorological parameters, excluding the effect of multicollinearity from Model B) are shown by light, medium, and dark gray solid lines, respectively. The vertical axis indicates the monthly RRD frequency. The predictive power increased from Model A to Model C, as the parameters were reduced.

Predicted onsets of each model, calculated using the leave-one-out cross-validation method, were compared with observed onsets by time course (Fig. 2). Predictive power improved from Model A to Model C, as parameters were reduced.

## Discussion

In this study, we analyzed the monthly and seasonal variations in RRD frequency as well as the association of meteorological factors and primary RRD frequency. The key findings of our study include the following: (1) no significant differences were found within monthly or seasonal variations of RRD frequency; (2) low atmospheric pressure and high stability (low ADV) of the seven meteorological parameters significantly affected the increase in RRD frequency; and (3) RRD frequency can be predicted based on specific meteorological parameters with excellent reliability and predictability using a Poisson regression model.

This study, performed in Kashiwa, Japan, showed that the highest RRD frequency was observed in summer and lowest in winter (Fig. 1). However, no significant differences were observed within monthly or seasonal variations in RRD frequency. Previous studies have also investigated monthly and/or seasonal variations in RD frequency in different geographical regions, reporting contradictory results^15,17–19,21,23,24,28^. Several studies from Lebanon^17^, Finland^28^, Spain^21^, India^19^, and Taiwan^18^ have reported a significant increase in RD frequency during warmer seasons (spring and/or summer) compared with colder seasons (fall and/or winter). In contrast, two studies from Turkey^24^ and Croatia^23^ reported no significant seasonal variations, and one study from Kuwait^15^ reported a significant increase in the winter. Although the underlying cause of these seasonal variations has not been elucidated, several hypotheses have been suggested. One hypothesis is that ultraviolet light, which is abundant in summer, heats the vitreous and retina, induces posterior vitreous detachment, and results in RD^18^. Another hypothesis attributes changes in RD frequency to the seasonal change in behavior patterns (i.e., physical activities associated with meteorological factors^14^ and frequency of eye rubbing^17,29^). In summary, analysis of monthly and seasonal variations is insufficient to explain the association between meteorological factors and RD frequency, as these parameters may vary across regions.

To explain this association more precisely, we performed univariate analyses between 36 meteorological parameters and RRD frequency, identifying eight meteorological parameters that had significant negative coefficients. Among these, seven parameters could be categorized as ADVs. Although previous studies have also analyzed the association between meteorological parameters and RD frequency^15–18,22,23^, they have reported inconsistent results^15–18,22,23^. Some studies reported that RD frequency was associated with sunlight exposure, ambient temperature, and atmospheric pressure^15,16,18,22^, whereas two studies reported no correlation with ambient temperature, relative humidity, precipitation, or sunlight exposure^17,23^. In our study, we observed a negative correlation between atmospheric pressure and RD frequency, as previously reported^18^. The correlation between ambient temperature and RD frequency is also controversial: we observed no correlation, which was similar to some previous findings^17,30^; positive correlation was observed in one study^18^; and a negative correlation was observed in another^15^. Although there is some consensus on the mechanisms that may result in the seasonal variation of RD frequency, conflicting findings have made it difficult to conclusively determine how meteorological parameters influence RD frequency. Interestingly, our study showed that seven meteorological parameters (ADV) showed a significant negative correlation with RRD frequency, suggesting that meteorological stability plays an important role in RRD frequency. We speculated that meteorological stability would lead to an increase in physical activity, which in turn would result in vitreous instability. Instead of attributing changes in RRD frequency to individuals or a set of meteorological parameters, we hypothesize that the effect of overall meteorological stability may better explain our findings.

We developed three types of multivariate regression models using several meteorological parameters to predict RRD frequency. The third model (Model C) showed excellent AICc, BIC, CV-VE, and sustained VE using only six meteorological parameters, indicating that it could be used for predicting monthly RRD frequency with an accuracy of 84.82% (95% CI 75.18–93.72%). Our findings strongly suggest that a multivariate regression model is highly effective for predicting RRD frequency.

Our study had some limitations owing to its single-center study design. Our data had a selection bias because our hospital medicated patients with RRD with surgery and studied patients who underwent surgery. Also, it was difficult to fully analyze the effects of confounders because this was an ecological study that analyzed aggregated data^31^. There was no control for individual level confounders including smoking, comorbidity such as diabetes, socioeconomic status, and other similar factors. Some non-meteorological factors, such as some annual events which affect lifestyle or physical activity, may affect seasonal variations in the number of patients with RRD as previously suggested^32,33^. Further large-scale, multicenter, and prospective studies are necessary to establish a precise multivariate regression model that can predict monthly and daily RRD frequency.

In conclusion, this was the first study to investigate the association between meteorological parameters and RRD frequency in Japan. Our findings showed that specific meteorological parameters such as low atmospheric pressure and high meteorological stability have a significant correlation with high RRD frequency. In addition, we established the validity of our multivariate regression model (using six meteorological parameters) for the prediction of RRD frequency.

## Data Availability

The datasets generated and/or analyzed for this study are available from the corresponding author upon reasonable request.
